# Self-Perceived Quality of Life in Spanish-Speaking Women with Autism Spectrum Disorders

**DOI:** 10.1007/s10803-021-05400-2

**Published:** 2021-12-25

**Authors:** Paula Morales Hidalgo, María Merino Martínez, Angélica Gutiérrez González, Lola Garrote Petisco, Carol Amat Forcadell, Cynthia I. D’Agostino, Laura Pérez de la Varga

**Affiliations:** 1 Research Group on Nutrition and Mental Health (NUTRISAM), Research Center for Behavioral Assessment (CRAMC), Department of Psychology, University Rovira i Virgili, Tarragona, Spain; 2 Asperger- ASD Association of Camp de Tarragona, Tarragona, Spain; 3 Science of Education Department, Specific Didactics Department, Universidad de Burgos, Burgos, Spain; 4 Burgos Autism Association, Burgos, Spain; 5 Department of Social Anthropology, Basic Psychology Public Health, Universidad Pablo de Olavide, Sevilla, Spain; 6 Sevilla Autism Association, Sevilla, Spain; 7 Centro de Evaluación, Diagnóstico e Intervención (CEDIN), Valencia, Spain; 8 Maresme Health Consortium, Mataró Hospital, Mataró, Spain; 9 Gabinet d’Especialitats Mèdiques Associades Mataró (GEMASI), Mataró, Spain; 10 Department of Psychology and Pedagogical Sciences, Universidad CAECE, Buenos Aires, Argentina; 11 Pediatrics Service, Hospital de Clinicas José T. San Martín, Buenos Aires, Argentina; 12 Science of Education Department, Universidad de Burgos, Burgos, Spain; 13 Spanish Association of Autism Professionals (AETAPI), Cadiz, Spain

**Keywords:** Wellbeing, Autism spectrum disorders, Women, Adults, Personal Wellbeing Index

## Abstract

Perceived personal wellbeing tends to be lower in individuals with autism spectrum disorders (ASD), especially in the case of women. To develop the present study, the Personal Wellbeing Index (PWI-A) was administered to a Spanish-speaking sample of women with ASD (N = 401) and self-diagnosed ASD (N = 343), women without ASD (N = 373) and men with ASD (N = 65) to compare their self-perceptions. Women with ASD showed significantly lower wellbeing rates than women in the control group for the total PWI-A and across all the domains, and there were no significant differences when compared with self-diagnosed women with ASD and men with ASD. Autism explained between 24 and 26% of the decline in the PWI-A total score, with life achievement, future security, safety and relationships being the domains most highly correlated with the total. These findings are an effective contribution to improving diagnosis and professional intervention in women with ASD.

## Introduction

### The Concept of Self-Perceived Quality of Life in People with ASD

Autism spectrum disorder (ASD) is a neurodevelopmental disorder characterized by social communication and interaction and the presence of restricted, repetitive or stereotyped patterns of behavior and interests (American Psychiatric Association, 2013). It involves broad impairments in social cognition, intersubjectivity, executive functioning and information processing (Hobson, 2019; Mundy & Hogan, 1994; Velikonja et al., 2019) and is defined by great phenotypic heterogeneity depending on genetic, environmental, cognitive and social variables (Cantio et al, 2016; Shea et al., 2013; Williams, 2018). In the everyday life of people with ASD these characteristics are related to greater or lesser skills in functionality, especially in the development of communication and social relationships, autonomy or adaptive behavior, and academic or professional performance.

Research on ASD has traditionally been carried out from an androcentric perspective. For over a decade, mental health professionals have perceived the need to improve their knowledge of ASD specificity in females in order to achieve a better characterization of the disorder and facilitate early detection, diagnosis and intervention. The female phenotype is characterized by greater internalization and camouflage behaviors than in males, who usually show less awareness of their own characteristics (Dean et al., 2017; Jamison & Schuttler, 2015; Solomon et al, 2012). This often leads to a delay in ASD diagnosis for women and increases the vulnerability of autistic women to psychopathological comorbidities or co-occurrent problems such as mood disorders, self-injuries or suicidal behavior, and problems related to self-care and eating (Bargiela et al., 2016; Calderoni et al., 2015; Van Wijngaarden-Cremers, 2019). In addition to internalization, the desire to please, the development of social compensation mechanisms through copying, imitation and self-training, and late or no access to mental health services have a direct impact on women’s mental health and wellbeing (Duvekot et al., 2017).

Due to the lack of mental health protocols for women with ASD, we consider it particularly important to study personal wellbeing as a dimensional approach to assessing quality of life (QOL). Although there is no consensus on the subject, QOL is considered a multifaceted construct that involves a continuous gradual process of interaction of multiple factors including personal, social, family, economic and environmental aspects (Cummins & Nistico, 2002; Schalock, 2000). It involves the way a person experiences their own life meaningfully, the individual’s sense of living with a purpose regardless of expected adversities, and the understanding of their life through a feeling of general wellbeing (King et al., 2006). These perceptions are based on aspects of life such as standard of living, health, achievements, present and future security, interpersonal and relationships, inclusion in the community and spirituality (Cummins et al., 2002). Other definitions suggest that affection, resilience, personality (La Placa et al., 2013), social belonging, optimism and self-regulation (Wassell & Dodge, 2015) also underlie a successful perception of oneself. Accessing and keeping a job, academic achievement, establishing positive loving relationships and maintaining a good state of health over time are also important (Helliwell et al., 2018; Lyubomirsky et al., 2005). In general terms wellbeing has often been identified or associated with happiness. In the context of ASD, some authors emphasize the importance of addressing QOL in people with ASD from the perspective of their own perception, being aware of the negative impact that may be involved in forcing people with ASD to fit into the neurotypical concept of happiness (Bishop-Fitzpatrick et al., 2017; Bishop-Fitzpatrick et al., 2017; Vermeulen, 2014). These researchers also point out the importance of translating the QOL concept into more specific and concrete indicators when it is assessed in this population.

In the study of QOL in autism, the information concerning the Spanish-speaking population is scarce. The literature includes the intersections between ASD and sociocultural variables, showing the influence of culture in the diagnosis of autism and its treatment (Burkett et al., 2007; Ennis-Cole et al., 2013; Perepa, 2019) and also in prevalence studies (Grinker et al., 2011). Other research delves into cross-cultural differences by analyzing individuals from different nations, such as Korea (Kang-Yi et al., 2013), Israel, South Korea, United Kingdom and United States (Matson et al., 2011) or Lebanon (Obeid et al., 2015), and more studies highlight the relevance of cultural analysis in the approach to autism as a field of analysis (Cascio, 2015; Daley, 2002; Dyches et al., 2004; Kim, 2012; Ravindran & Myers, 2012), although none of them focuses specifically on Spanish-speaking contexts.

### Personal Wellbeing and Self-Perceived Quality of Life in Women with ASD

Recent studies have indicated that adults with ASD are more vulnerable to a variety of negative life experiences including employment difficulties, financial problems and domestic abuse, all of which are related to anxiety, depression and a less satisfying life than that of adults without this condition (Griffiths et al., 2019), leading to a significantly lower perceived QOL throughout the lifespan of adults with ASD (Egilson et al., 2017; van Heijst & Geurts, 2015). Although there are value-based studies on the QOL and life experiences of adults with ASD, none of these included Spanish-speaking participants and there are very few studies that focus exclusively on women or that compare both sexes.

In this sense, Baldwin and Cosley (2015) examined the needs reported by 82 Australian adult women with high-functioning ASD and noted that, although most analyses found no gender differences in connection with diagnosis, health, education, work, and social and community activities, participants highlighted the diverse and complex challenges they faced as women, including high levels of comorbidity with mental health disorders, unmet support needs in education and work settings, and social exclusion and isolation. The Autism in Pink project (2014) –which became a baseline study on the needs and QOL of women with ASD, stemming from which discussion groups were held and the Personal Wellbeing Index Adult (PWI-A) tool administered (International Wellbeing Group, 2013)– showed a lower perception of QOL in women with ASD in all the personal wellbeing indices analyzed, both in comparison to men with ASD and women without ASD. The biggest differences were found in the indicators for health and wellbeing, life achievements, social relations and inclusion in the community, but there were no significant differences in the levels of general QOL and current and future security. Neither were any significant differences found in perceived satisfaction with spirituality, which is important since spirituality and religion have proven to be an important aspect for many people with ASD, as it relates to their quality of life, shapes their view of disability and gives it a positive meaning (Cwik, 2021; Liu et al., 2014). In addition, when analyzing possible predictors of QOL on the basis of the four domains described by the World Health Organization –physical (including pain, energy), psychological (including positive and negative feelings, concentration), social (personal relationships, friends, sexuality) and environment (including safety, partaking in leisure activities and monetary resources)– Mason et al. (2018) found three main characteristics that may be predictive of a low QOL in this population: being female, having an associated mental condition and the severity of the autism symptoms.

Women with ASD therefore seem to be more likely to experience a lower QOL than men with ASD, especially when they have psychopathological comorbidities or, as suggested by McGillivray and Evert (2018), experience everyday stress factors such as social events and uncertainty. This is reason enough to carry out an in-depth study of the needs, trajectories and particularities of women with ASD in order to establish more precise preventive and therapeutic programs (Cage et al., 2017; Green et al., 2019). In turn, the increased presence of internalized symptoms in adolescents and women with ASD (Green et al., 2019; Oswald et al., 2015) can remain undetected until they become chronic, thereby impacting their adaptability and QOL. Women also have to cope with further health risks and other medical problems related to organic, hormonal and menstrual functioning (DaWalt et al., 2021; Steward et al., 2018) and complications deriving from pregnancy and motherhood (Sundelin et al., 2018; Turner, 2017). Exploratory and descriptive studies based on their experiences may help to more specifically identify their needs and guide future research. Some areas to look at in more detail could be gender identity and social relationships (Kanfiszer et al., 2017), the diagnostic process, symptom management and understanding, plus the impact of ASD symptoms on personal and work relationships (Haney & Cullen, 2017), prevention of abuse (Pecora et al., 2016; Roberts et al., 2015) and suicide (Moseley, 2019; Bringmann & Maidman, 2019). Cultural expectations and gender stereotypes also have a specific influence on the QOL of women with ASD (Draaisma, 2009; Fahs, 2020; Leedham et al., 2020; Moseley et al., 2020). Milner et al. (2019) highlighted five issues to consider: their fit to the norms, potential barriers for women and girls with ASD, the negative aspects of ASD, the perspective of others, and the positive aspects of being autistic (Milner et al., 2019). Other core aspects in connection with the cultural role of women that a specific focus on women with ASD requires would involve challenges relating to aspects such as sexuality and motherhood and their impact on the sense of security (Gardner et al., 2016; Rogers et al., 2017). These studies were, mostly, phenomenological and narrative approaches that foregrounded the lived experiences of people with ASD and showed the broad field of study from qualitative perspectives. The inclusion of self-diagnosed individuals with ASD responds to the situation of difficulty that many encounter in obtaining a clinical diagnosis and is relevant to this study.

Taking all the above into account, the aim of the present study is to analyse the QOL of a large group of women with a diagnosis of ASD compared to men with the same diagnosis, women with self-diagnosis and women without ASD. We propose to analyse the similarities and differences in the perception of QOL of ASD women compared to their male and female control peers through a quantitative assessment in the shape of the PWI-A. The data about women are complemented by several meaningful items about life experiences and personal thoughts enabling us to obtain objective indicators relating to the eight domains described by Cummins et al. (2003) in the PWI-A. This analysis will allow us to identify the impact that being a woman and having ASD has on perceived QOL and thus on personal wellbeing. On the basis of the previous literature on gender and ASD, we hypothesize that lower levels of personal wellbeing will be perceived by women with ASD compared to their counterparts with self-diagnoses and without ASD and also with males with ASD.

## Method

### Study Design and Procedure

The “Women and ASD: invisibility or difference” research project is a cross-sectional study by a research group from the Spanish Association of Autism Professionals (Asociación Española de Profesionales del Autismo, AETAPI), the main aim of which is to investigate the psychopathological characteristics and QOL in girls, adolescents and women with ASD. Data were obtained between 2015 and 2018 by way of an online survey distributed by the researchers to a Spanish-speaking population supported by professionals and organizations working together with people with ASD, who allowed us to deliver the questionnaire on-line to the target population.

The survey gathered information about diagnostic, comorbidities and sociodemographic characteristics. It also included the Personal Wellbeing Index-Adult (PWI-A) and several indicators specifically designed for the autistic female population in each PWI domain. An interactive two-round expert consensus process based on insights from the Autism in Pink Project (National Autistic Society) was used to select these indicators. The study was approved by the Ethical Committee concerning Research into People, Society and the Environment at the Rovira i Virgili University (CEIPSA-2021-PR-0007).

As far as the community involvement is concerned, this study has arisen from the cooperative work of members of an association of professionals dedicated to ASD, AETAPI. Also, professionals and organizations in the field of ASD helped us distribute the survey.

### Instruments

The online survey comprised a set of questions about clinical and sociodemographic characteristics such as age, sex and country of residence. Participants with ASD were asked about diagnostic categories and the presence of psychopathological comorbidities in terms of ICD or DSM classifications. In addition, they supplied the date on which the diagnosis was obtained and the type of professional referring. Several dichotomous questions about past and current ASD symptoms were included in order to corroborate previous diagnoses. We specifically asked about impairments or delays in key developmental milestones, social and communication difficulties, repetitive and stereotyped behavior patterns, sensory disturbances, emotion management, compensatory social strategies and emotion regulation. The comparison group without ASD was also asked for information regarding the presence of psychopathological problems and the type of professional who performed the assessment.

The Personal Wellbeing Index-Adult (PWI-A; International Wellbeing Group, 2013) was used in the present study to assess participants’ current life satisfaction. The PWI-A emerged from the Comprehensive Quality of Life Scale (ComQol; Cummins et al., 1994) and is based on Cummin’s homeostasis theory of wellbeing (Cummins & Nistico, 2002). It has been designed to be a valid cross-cultural questionnaire. The Spanish version, adapted by Rodriguez-Blazquez et al. (2011) and Forjaz et al. (2011), has proven to be unidimensional with good internal reliability (α = 0.88). The PWI-A provides a total QOL score and separate scores for each of the following domains: standard of living, health, achieving in life, relationships, safety, community-connectedness and future security, spirituality or religion. The answer format consists of an end-defined scale from 0 (no satisfaction at all) to 10 (complete satisfaction) in each domain to estimate the subjective perception of QOL, without having more specific indicators. Following the authors’ suggestion, each domain was analyzed separately at statistical level and the seven primary domains were grouped to obtain an average score for subjective wellbeing.

In addition to the PWI-A, a set of dichotomous items was included for each wellbeing domain. The standard of living domain allowed us to explore the presence of positive or negative thoughts like “I get things just as easily as anyone else” or “I would like my life to be different”. The health domain explored illness and personal habits involving exercise, diet, hygiene, clothing and sexuality. Achieving in life included items relating to educational level, employment and economic independence, and coping with life obstacles. Relationship items assessed social interaction in several contexts, sexual orientation and the occurrence of harassment and abuse, while community-connectedness explored the participant’s profile of belonging to face-to-face or virtual groups. Safety and future security items asked about the certainty of basic security needs involving food, health, shelter, and social and personal protection. The last set of items explored spirituality and religion feelings and thoughts.

### Sample

The sample for the present study comprised 1182 participants from Spanish-speaking populations with an average age of 33.08 years (SD 9.12) and was recruited by means of an on-line survey. The sample was divided into different groups including women with ASD (formally diagnosed N = 401 and self-diagnosed N = 343), women without ASD (N = 373) and a small comparison subgroup of men with ASD (N = 65). The inclusion criteria for all the groups were being over the age of eighteen and having sufficient cognitive and language skills to be able to answer the questionnaire. In the ASD groups, formal diagnosis by a specialized professional was required. Participants with ASD and other comorbidities were not excluded, but this information was specifically requested. Only in the group of women with ASD were responses accepted under the premise of “self-diagnosis” due to the difficulties these women faced in obtaining a diagnosis. They had to state explicitly that they did not have a formal diagnosis from any professional but were able to access the questionnaire, and this variable meant that the groups with and without formal diagnosis could be clearly differentiated. In the control group, responses from adult women without ASD or other pathologies were accepted. Exclusion criteria were considered not being a relative of a person with ASD and not working in the field of ASD. The sociodemographic characteristics of the sample are described in Table 1 and analysed in greater detail in the results section.

**Table 1:** Sociodemographic characteristics of the sample

	*Women sample*				*Men subsample*	
	ASD women N = 401	Self-diagnosed ASD N = 343	Non-ASD N = 373	*p*_*a*_	ASD men N = 65	*p*_*b*_
Current age	33.11 (8.88)	35.28 (10.20)	34.36 (10.65)	0.009*	32.91 (9.96)	0.885
Nationality						
Spain	79 (19.70)	74 (21.57)	312 (83.65)	**0.001**	28 (43.07)	**0.001**
Other European countries	9 (2.24)	6 (1.75)	7 (1.87)		0 (.00)	
North America	5 (1.25)	3 (.88)	0 (.00)		0 (.00)	
Central and South America	308 (76.81)	260 (75.80)	54 (14.40)		37 (56.93)	
Educational level						
Primary education	63 (15.71)	53 (15.45)	73 (19.47)		19 (29.23)	
Secondary education	122 (30.42)	102 (29.74)	63 (16.80)	**0.001**	10 (15.36)	**0.006**
Higher education	216 (53.87)	188 (54.81)	239 (63.73)		36 (55.39)	
Currently employed	189 (47.13)	140 (40.81)	222 (59.20)	**0.001**	10 (15.38)	**0.001**
Current partner	200 (49.88)	262 (76.39)	233 (62.13)	**0.001**	14 (21.54)	**0.001**

Categorical data was presented in frequencies and percentages (N, %) and analyzed through chi-squared test. Continuous data was presented in means and standard deviations (M, SD) and analyzed through W-ANOVA (women samples) and t-test for independent samples (women versus men with ASD). Significant differences in bold (*p* ≤ 0.01)

*P*_a_ compared the three women samples while *P*_b_ compared women and men with ASD.0

*Post-hoc analysis revealed differences between ASD and self-diagnosed ASD (*p* = .007)

### Data Analysis

Data analyses were performed using IBM SPSS 25. The internal reliability of the instrument and the dichotomous items about wellbeing was evaluated using Cronbach’s alpha. In the present sample, the reliability of the PWI-A and the dichotomous wellbeing items were α = 0.91 and α = 0.83, respectively. The reliability of the items assessing present and past symptoms of ASD was α = 0.89. Sociodemographic and clinical characteristics were provided through descriptive statistics, and comparisons were made by means of chi-squared and t-test for independent samples, depending on the dependent variable to be processed. Levene’s test was used to assess variance homogeneity in t-test. In terms of QOL (PWI-A scores), the groups were compared using Welch’s corrected ANOVA (W-ANOVA) and Tamhane’s T2 t-test for post-hoc comparisons and on the assumption that differences in sample size were likely to result in heterogeneity of variances. Pearson correlations were also carried out between the different dimensions of the PWI-A and the total score. Taking into account the findings of the t-test analyses and the Welch’s corrected ANOVA, adjusted and unadjusted multiple linear regression models using the enter method were used to explore the effect of ASD diagnoses, anxiety and depression comorbidities and sex in the subjective perception of QOL. Nationality and age variables were excluded from the analysis as they showed a very low correlation with the dependent variable. Due to the multiple comparisons, a Bonferroni correction was applied to control for the probability of committing a type I error.

## Results

### Sociodemographic and Clinical Characterization of the Sample

Sociodemographic characterization of the sample is presented in Table 1. Women with ASD and self-diagnosed ASD showed lower educational levels than their female counterparts, as well as lower rates of current employment and romantic partners. In contrast, these ratios were higher in women with ASD in comparison to men. The self-diagnosis group of women presented an average age slightly higher than the rest.

Based on the clinical information given by the women with ASD, diagnoses were mainly provided by a psychiatrist (70.8%), while 26.4% indicated that they had requested an assessment by a psychologist or a mixed team of professionals (including psychiatrist, psychologist and neurologist) and 2.7% by other health or education professionals. Asperger disorder was the most frequent diagnostic category (70.8%), followed by ASD (23.7%) and to a lesser extent pervasive developmental disorders (PDD, 2.8%). Psychopathological comorbidities were present in 77.6% of cases, with anxiety disorders (45.9%) and depressive disorders (38.7%) being the most prevalent. Comorbidities were also frequent in the self-diagnosis group (61.5%), although significantly lower than in the ASD group (*p* = 0.001), with high rates of anxiety disorders (39.7%) and depressive disorders (31.2%). As far as the men with ASD group was concerned, diagnoses were mostly provided by a psychiatrist (76.92%), psychologist or mixed team of professionals (20.00%) or other health or education professionals (3.08%). The main diagnostic category was Asperger disorder (76.92%), followed by ASD (23.07%), autism disorder (4.62%) and pervasive developmental disorders (3.08%). Psychopathological comorbidities were less common in males (43.08%), who presented mainly depressive disorders (27.69%), anxiety disorders (26.15%) and attention deficit and hyperactivity disorder (23.08%). In comparison with previously presented data on women with ASD, no sex differences were found in terms of ASD category or referral professional, but women with ASD showed higher rates of comorbidity than men with ASD (*p* = 0.012).

Because of the inclusion of a group of self-diagnosed ASD women in the study and in order to give greater validity to further results, we have compared the answers of the three groups of women (ASD, self-diagnosed ASD and control) for the items relating to socio-communicative impairments and repetitive patterns of behavior in childhood, which are core features of ASD. In order to explain these results, the differences between the three groups of women are described as total and those between women with formal and self-diagnosed ASD as partial. Comparisons were performed by means of chi-squared test. At a social-communicative level, women with ASD and self-diagnosed ASD exhibited significant difficulties in social initiation (ASD: 66.3%, self-diagnosed ASD: 66.8%, Control: 17.1%; *p*_*total*_ = 0.001 and *p*_*partial*_ = 0.901), the development of reciprocal friendships (ASD: 36.4%, self-diagnosed ASD: 36.7%, Control: 5.3%; *p*_*total*_ = 0.001 and *p*_*partial*_ = 0.927), and the experience of social exclusion (ASD: 69.8%, self-diagnosed ASD: 60.9%, Control: 15.7%; *p*_*total*_ = 0.001 and *p*_*partial*_ = 0.011). Similarities between women with ASD and self-diagnosed ASD were also observed in the use of coping strategies when faced with social situations. Both groups tended to plan conversations, analyze social situations posteriori and repetitively and study human behavior through books, films and series. In contrast, women without ASD did not usually need specific strategies to manage relationships or they turned to others to solve social doubts and concerns. In the area of repetitive patterns of behavior, in the groups with ASD there was a notable presence of balancing behavior to deal with frustration (ASD: 23.4%, self-diagnosed ASD: 12.5%, Control: 0.3%; *p*_*total*_ = 0.001 and *p*_*partial*_ = 0.001), perfectionist behaviors at school (ASD: 25.4%, self-diagnosed ASD: 22.2%, Control: 6.1%; *p*_*total*_ = 0.001 and *p*_*partial*_ = 0.097) and sensory alterations at the level of both hypersensitivity (ASD: 70.6%, self-diagnosed ASD: 66.2%, Control: 9.6%; *p*_*total*_ = 0.001 and *p*_*partial*_ = 0.198) and hyposensitivity (ASD: 13.0%, self-diagnosed ASD: 13.4%, Control: 6.1%; *p*_*total*_ = 0.002 and *p*_*partial*_ = 0.858). As for executive functioning, the groups with ASD and self-diagnosed ASD showed greater difficulty in time management (ASD: 29.2%, self-diagnosed ASD: 23.3%, Control: 0.3%; *p*_*total*_ = 0.001 and *p*_*partial*_ = 0.071) and processing speed (ASD: 25.4%, self-diagnosed ASD: 22.2%, Control: 6.1%; *p*_*total*_ = 0.001 and *p*_*partial*_ = 0.296) in childhood.

### Subjective Perception of Wellbeing in Women with ASD and Comparison Groups

As there was a different distribution of nationalities among the participants in each group, it was considered that this may lead to differences in the self-perceived QOL. A W-ANOVA revealed intra-group invariance by nationality in the subjective perception of QOL on both the total scale and the different subscales. The comparison between groups as regards QOL was therefore performed without this differentiation (Table 2).

**Table 2:** Differences in subjective perception of quality of life between groups

	ASD women_a_		Self-diagnosed ASD women_b_		Non-ASD women_c_		ASD men_d_		*p*	_ab_	_ac_	_ad_	_bc_	_bd_
Total PWI-A	5.01	(2.09)	5.00	(2.10)	7.35	(1.41)	5.67	(2.08)	**0.001**	0.999	**0.001**	0.108	0.111	0.282
Standard of living	5.02	(2.49)	4.88	(2.57)	7.34	(1.62)	5.55	(2.47)	**0.001**	0.970	**0.001**	0.502	**0.001**	0.251
Health	5.31	(2.70)	5.25	(2.60)	7.21	(1.94)	6.06	(2.64)	**0.001**	0.999	**0.001**	0.202	**0.001**	0.144
Achieving in life	5.39	(2.62)	5.23	(2.62)	7.40	(1.76)	6.02	(2.45)	**0.001**	0.959	**0.001**	0.326	**0.001**	0.123
Relationships	4.72	(2.63)	4.69	(2.49)	7.77	(1.61)	5.48	(2.30)	**0.001**	0.999	**0.001**	0.103	**0.001**	0.084
Safety	5.48	(2.86)	5.57	(2.71)	7.26	(1.92)	6.17	(2.67)	**0.001**	0.999	**0.001**	0.308	**0.001**	0.471
Community-connectedness	4.31	(2.67)	4.41	(2.63)	7.51	(1.78)	5.12	(2.91)	**0.001**	0.997	**0.001**	0.207	**0.001**	0.347
Future security	4.82	(2.82)	4.94	(2.77)	6.92	(1.97)	5.31	(2.85)	**0.001**	0.999	**0.001**	0.907	**0.001**	0.916
Spirituality or religion	6.21	(3.10)	6.01	(3.02)	7.31	(5.40)	6.72	(3.21)	**0.001**	0.943	**0.001**	0.797	**0.001**	0.477

Data presented in means (M) and standard deviations (SD). Analyses were performed by means of W-ANOVA and significant differences in bold *p* ≤ 0.01. Lowercase letters indicate comparisons between groups: a (women with ASD), b (self-diagnosed women with ASD), c (non-ASD women) and d (men with ASD)

**Fig. 1:**
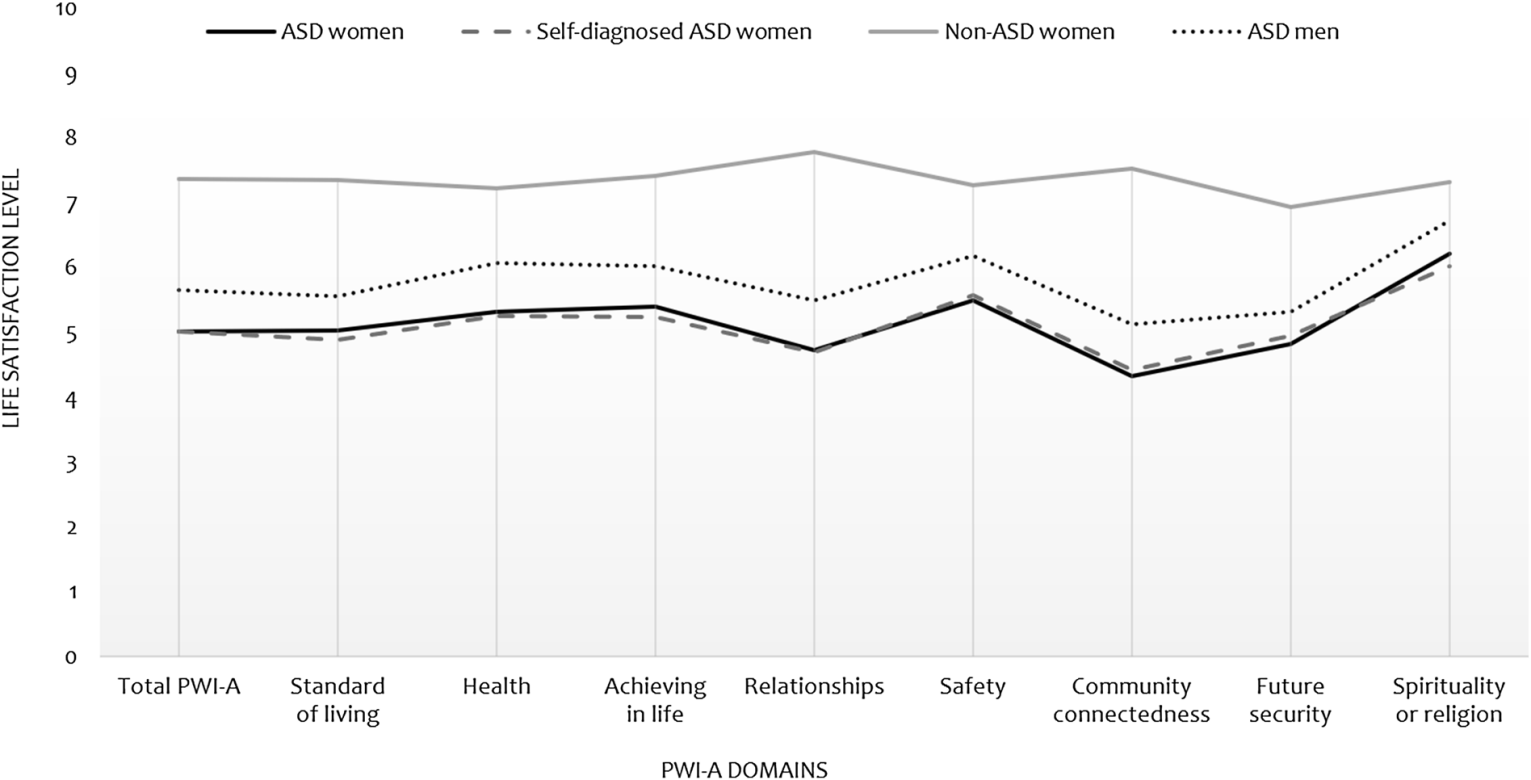
Profile of mean scores by groups across the different quality of life domains. *PWI-A* Personal Well-being Index Adults; *ASD* autism spectrum disorders; *Non-ASD* absence of ASD diagnosis

We found significant differences between groups for all PWI-A dimensions and the total score by means of a W-ANOVA. Post-hoc analyses revealed that the women with ASD diagnoses and the self-diagnosed had the lowest perception of subjective life quality, with no statistically significant differences between them and an almost overlapping score profile (Fig. 1). The lowest scores among women with ASD were in relation to relationships, community-connectedness and future security domains, while the highest score was for the spirituality domain. Self-diagnosed women with ASD also reported low satisfaction with standard of living. Both groups differed significantly from the without ASD group (*p* = 0.001), whose scores were around two points higher. No differences were found when compared with males with ASD, although this group scored slightly higher in PWI-A. The most affected domains in males were also related to relationships, community-connectedness and future security. Figure 1 shows how all the groups with ASD, unlike the non-ASD, exhibit a largely identical profile of perceived QOL, and that it was the women who presented the lowest perception of wellbeing.

### PWI-A Domains and Main Quality of Life Indicators Among Women with ASD

In global terms, correlations between the domains and the total PWI-A score were significant (*p* = 0.001) and ranged from moderate to very high, with achieving in life (Pearson’s *r* = 0.860), future security (*r* = 0.868), safety (*r* = 0.843) and relationships (*r* = 0.800) being the most associated domains. As regards life achievements, women with ASD reported significantly less economic independence (ASD: 30.7%, self-diagnosed ASD: 29.7%, control: 51.7%; *p* = 0.001), poorer achievement of personal goals (ASD: 13.9%, self-diagnosed ASD: 16.6%, control: 25.3%; *p* = 0.001) and greater belief in having bad luck than the controls (ASD: 22.0%, self-diagnosed ASD: 16.9%, control: 0.0%). At the same time they reported having been able to overcome many difficulties in their lives (66.8%, 51.0% and 49.6% respectively). The biggest difference between them and their peers in relationships was found in the items “The relationship with the family is good” (ASD: 29.9%, self-diagnosed ASD: 26.5%, control: 57.6%; *p* = 0.001), “I barely have relationships but I don't want them” (ASD: 36.7%, self-diagnosed ASD: 34.7%, control: 2.4%; *p* = 0.001) and “I have had dangerous experiences because I trusted people” (42.4%, 41.7% and 4.3% respectively). Safety and future security were also very important aspects in the perception of wellbeing in women with ASD. They were more likely to often feel fearful (ASD: 42.1%, self-diagnosed ASD: 49.0%, control: 12.0%; *p* = 0.001) and unprotected by their families (ASD: 20.2%, self-diagnosed ASD: 24.2%, control: 4.3%; *p* = 0.001). They also considered themselves much less capable of living on their own (ASD: 41.4%, self-diagnosed ASD: 42.2%, control: 80.3%; *p* = 0.001) and overcoming life’s challenges (37.7%, 37.3% and 62.9% respectively; *p* = 0.001).

Moderate to high correlations were found between the PWI-A and community-connectedness (*r* = 0.755), health (*r* = 0.744), standard of living (*r* = 0.627) and spirituality or religion (*r* = 0.592). As far as community is concerned, women with ASD have a low participation in generic leisure groups (ASD: 17.7%, self-diagnosed ASD: 13.7%, control: 46.1%; *p* = 0.001), feel misunderstood by groups (ASD: 40.2%, self-diagnosed ASD: 42.6%, control: 6.4%; *p* = 0.001) and tend to limit themselves to internet relationships (ASD: 30.2%, self-diagnosed ASD: 27.7%, control: 0.5%; *p* = 0.001). As regards the health domain, significantly more women with ASD (ASD: 29.4%, self-diagnosed ASD: 24.5%) reported being on pharmacological treatment compared to their peers without ASD (3.7%; *p* = 0.001). They were also more likely to have an unbalanced diet (ASD: 46.1%, self-diagnosed ASD: 44.6%, control: 68.0%; *p* = 0.001) and not be engaged in any sporting activity (ASD group: 49.1%, self-diagnosed ASD group: 42.3%, control: 19.5%; *p* = 0.001). Of the specific indicators included in the standard of living domain, women with ASD showed the highest rates of dissatisfaction with life. The items that best defined their thoughts or feelings were “I think life is difficult” (ASD: 56.9%, self-diagnosed ASD: 55.1%, control: 20.5%; *p* = 0.001) and “I would like my life to be different” (ASD: 48.1%, self-diagnosed ASD: 53.9%, control: 18.9%; *p* = 0.001). Finally, regarding spirituality or religion, they often feel that their beliefs are poorly respected (ASD: 24.2%, self-diagnosed ASD: 21.3%, control: 40.3%; *p* = 0.001) even though spiritual feeling is very important for many of them (ASD: 43.1%, self-diagnosed ASD: 34.7%, control: 20.0%; *p* = 0.001).

### Association Between ASD, Psychopathological Comorbidities, Life Experiences and Wellbeing

Considering the lack of differences in wellbeing perception in terms of nationality, the variability in the subjective perception of QOL may be related to ASD diagnoses and/or associated problems. Unadjusted and adjusted regression models were performed on the whole sample in order to observe the effect of sex, ASD and the most frequent psychopathological comorbidities—anxiety and depression disorders—on the global perception of QOL. An ASD diagnosis by itself explains 24% of the total variability (*β* = − 2.29, *F*_*(1,1181)*_ = 369.10, *p* = 0.001). In the adjusted model the explained variability was slightly higher (*CR*^2^ = 0.261, *F*_*(4,1178)*_ = 105.37; *p* = 0.001) and ASD (*β* = − 2.03, *t* = 30.19, *p* = 0.001), depression (*β* = − 0.727, *t* = − 4.85, *p* = 0.001) and sex (*β* = − 0.297, *t* = − 2.43, *p* = . 015) were significantly related to decline in the PWI-A, while the presence of anxiety disorder turned out to be non-significant (*β* = − 0.153, *t* = − 1.05, *p* = 0.293). The results were very similar in the female subsample, where the unadjusted model of ASD explains 26% of the variability (*β* = − 2.35, *F*_*(1,1116)*_ = 382.56, *p* = 0.001), while in the adjusted model ASD and depression, but not anxiety, explain 27% of the variability (*F*_*(3,1114)*_ = 139.37; *p* = 0.001). In the women with formal or self-diagnosed ASD, the presence of comorbidities contributes to a worsening perception of QOL. The average PWI-A total scores were 5.26 (standard deviation, SD, 2.11) for those without comorbid mood disorders, 5.25 (SD 2.17) for those with comorbid anxiety disorder, 4.90 (SD 1.92) for those with comorbid depressive disorder and 4.37 (SD 1.96) for those with comorbid anxiety and depressive disorders. Significant differences were found only between females with ASD and no comorbid mood disorders and ASD with comorbid anxiety and depressive disorders (*p* = 0.001).

## Discussion

As far as we know, this is the first study to provide information about perceived QOL in Spanish-speaking population by comparing women with formal and self-diagnosed ASD, men with ASD diagnoses and non-autistic women. It extends previous findings from the PWI-A in the Autism in Pink European project (Kenyon, 2014), which highlighted the importance of exploring perceptions and life trajectories in order to understand intersections between gender and ASD, but focused on a much smaller sample of women on the spectrum.

The self-diagnosed ASD group of women displayed manifestations compatible with ASD and similar to the clinical group when the psychopathological and psychosocial characteristics of both groups were compared. When focusing on core autistic symptoms, at a socio-communicative level we found difficulties in social initiation and socio-emotional reciprocity along with camouflaging and analytical strategies to cope with relationships and pragmatic communication. Regarding stereotyped behaviors and sensorial sensitivity, these two groups were also homogeneous in their distribution. Subsequent studies are needed to continue exploring similarities, which would help with detection, and also those subjective experiences that may identify a lifelong milestones profile compatible with an ASD diagnosis. Judging by the previous literature, there is a lack of research using samples of self-diagnosed women with ASD. Understanding this group could increase our knowledge of underdiagnosed and misdiagnosed women on the spectrum. Although several studies have confirmed the delay in diagnosing women with ASD and the existence of barriers including camouflaging and clinician stereotypes, there is a lack of research on this “lost generation” group (McDonald, 2020). More findings could help to define common and core features between self-diagnosed women and those with formal diagnoses.

As far as the subjective perception of QOL is concerned, we hypothesized that women with ASD would have the lowest levels of personal well-being compared to the rest of the groups. In the present sample women with formal and self-diagnosed ASD presented a lower QOL than the control women. Differences were not significant compared to men with ASD, although the mean scores were quantitatively lower. It is possible that this greater impact on personal wellbeing could be associated with a higher rate of mood disorders and other psychopathological comorbidities in women with ASD. McGillivray and Evert (2018) point to the greater vulnerability of women with ASD to stress factors, especially those linked to everyday and social events, anticipation and uncertainty, which could also lead them to have a lower perception of wellbeing. The emotional impact may also lead self-diagnosed women to develop comorbidities, despite the fact that social and communication difficulties have yet to be diagnosed. The difficulty women face in accessing diagnosis and adequate treatment can exacerbate this emotional distress and make them suffer unnecessarily for a long period of time (van Wijngaarden, 2017). In regard to health status, DaWalt et al. (2021) also found greater vulnerability among women with ASD to certain conditions such as nutritional disorders, sleep disorders, neurological and psychiatric disorders, endocrinal disorders and gastrointestinal disorders when compared with men.

The PWI-A profiles of formal and self-diagnosed ASD women overlap so much that they appear to form a single group with identical levels of suffering and impact. We expected to find higher impairment in the formally diagnosed group because we thought that previously diagnosed cases were more likely to be more severe or complex. However, the difficulty of women with ASD in obtaining a diagnosis might explain the certain equivalence between the two groups. In line with previous studies, the control subsample scores were generally higher in all significant areas of subjective perception (Cummins & Nistico, 2002), while women with ASD showed a significantly lower perception (Kenyon, 2014). In the general population, Diener (2000) stated that the adaptive capacity of individuals is closely related to their quality of life. The difficulties that people with ASD have with being flexible could imply that they do not readjust their perception or behavior in the same way in order to maintain a positive perception of their wellbeing. In addition, the presence of social and communication impairments in the ASD population would explain why these areas show the lowest QOL scores. The findings of the Autism in Pink project (Kenyon, 2014), which indicated that the most affected areas in women’s PWI-A were the impact on health and wellbeing, life achievements, social relations and inclusion in the community, are relatively close to our results. The differences between the two studies might be explained by differences in sample size and the mean age of the participants, which could influence their current concerns or obstacles in life. Our results highlight the impact on the relationships, community-connectedness and future security domains, which is congruent with the results obtained by Kanfiszer et al. (2017). These authors explored the perceived wellbeing and experiences in social relationships of women on the spectrum and pointed out the specific difficulties they face in social situations and, in particular, their sexual vulnerability, especially in childhood. According to Bargiela et al. (2016), there are two main elements involved in understanding the social difficulties of women with ASD. Firstly, women with ASD exhibit high vulnerability due to their being exposed to dangerous situations and feeling unprotected. Secondly, they have past experience of having felt unprotected by their families in different violent situations such as bullying or sexual abuse. Prior memories of traumatic events and feelings of insecurity affect women’s QOL. In this respect, women with ASD often report being aware that their naivety, excessive trust in others and inability to differentiate their intentions exposes them to dangerous situations. Unlike men on the spectrum, women become involved in groups more frequently, but at a high emotional cost due to the perception of not fitting in and a desire to maintain shorter social interactions, which undoubtedly affects their wellbeing.

Another factor that we have found to be associated with personal wellbeing is life achievements, an area in which there is a discrepancy between academic goals and access to equivalent job positions or the acquisition of personal and economic independence. Several factors such as pragmatic skills, executive functioning and the ability to manage their own money can play a significant role in this area. In our sample, women with ASD showed lower employment rates than women without ASD, despite showing a slightly higher educational level. Their employability rate was much higher than that of the group of men with ASD, which is in contrast to a large body of disability studies (Lonsdale, 1990; Vernon, 1997) and to the rate commonly reported in the ASD population (Taylor et al., 2019). In our sample, this result may be possibly due to women’s greater skills in adapting to context or differences in the levels of severity of ASD. In this respect, Helliwell et al. (2018) highlighted a strong association between employment and wellbeing, arguing that having a job improves subjective wellbeing by up to 30% compared to the unemployed in the general population. Also, in the ASD population employment has been related with wellbeing as well with economic independence, which leads to a greater possibility of independent living, participation in community settings and self-esteem (Stodden & Mruzeck, 2010).

The main limitations of this study are related to the sample and data collection. Because this was an online survey, it may include representation errors and biases in obtaining information. Age, diagnosis variables and desirability have been controlled to exclude those answers in the questionnaires that could be biased. Self-diagnosed women were not recruited for diagnosis after participation, since appropriate diagnostic guidance would be required. Cognitive skills and the severity of the autism phenotype have not been controlled in this study, and these variables should be taken into account in future studies.

In summary, it can be concluded from the data presented in this research that the QOL levels perceived by women with formal and self-diagnosed ASD are significantly lower than in women without ASD. A substantial overlap of lower PWI-A rates was found between the groups with ASD. However, no superadditive effect of psychopathological comorbidities such as anxiety or depression was found in the decrease of perceived wellbeing. ASD itself is a significant factor in the decrease in QOL, much more than other psychopathological or sociodemographic variables. Thus it is important to plan specific strategies for people with ASD that contribute to improving their wellbeing. Our findings open up a path to be explored, where further research will be needed to verify and complement these findings. In the expansion of these frontiers, it will be particularly important to include verification of the ASD diagnoses and the levels of severity, along with intellectual and language performance among other individual variables, in order to ensure the homogeneity of the samples and the reliability of the results.

## References

[CR1] American Psychiatric Association (2013). Diagnostic and statistical manual of mental disorders (DSM–5).

[CR2] Autism in Pink. (2014). http://www.autisminpink.net/. Retrieved 8 Nov 2021.

[CR3] Baldwin S, Costley D (2015). The experiences and needs of female adults with high-functioning autism spectrum disorder. Autism.

[CR4] Bargiela S, Steward R, Mandy W (2016). The experiences of late-diagnosed women with autism spectrum conditions: An investigation of the female autism phenotype. Journal of Autism and Developmental Disorders.

[CR5] Bishop-Fitzpatrick L, Mazefsky CA, Eack SM, Minshew NJ (2017). Correlates of social functioning in autism spectrum disorder: The role of social cognition. Research in Autism Spectrum Disorders.

[CR6] Bishop-Fitzpatrick L, Smith DaWalt L, Greenberg JS, Mailick MR (2017). Participation in recreational activities buffers the impact of perceived stress on quality of life in adults with autism spectrum disorder. Autism Research.

[CR7] Bringmann SA, Maidman PE (2019). Diagnosis of autism spectrum disorder in women with suicidality and characteristics of borderline personality disorder]. Tijdschrift Voor Psychiatrie.

[CR8] Burkett K, Morris E, Manning-Courtney P, Anthony J, Shambly-Ebron D (2007). African american families on autism diagnosis and treatment: The influence of culture. International Journal of Clinical Practice.

[CR9] Cage E, Di Monaco J, Newell V (2017). Experiences of autism acceptance and mental health in autistic adults. Journal of Autism and Developmental Disorders.

[CR10] Calderoni S, Fantozzi P, Balboni G, Pagni V, Franzoni E, Apicella F, Muratori F (2015). The impact of internalizing symptoms on autistic traits in adolescents with restrictive anorexia nervosa. Neuropsychiatric Disease and Treatment.

[CR11] Cantio C, Jepsen JRM, Madsen GF, Bilenberg N, White SJ (2016). Exploring ‘the autisms’ at a cognitive level. Autism Research.

[CR12] Cascio A (2015). Cross cultural autism studies, neurodiversity, and conceptualizations of autism. Culture, Medicine, and Psychiatry.

[CR102] Cummins RA, Eckersley R, Pallant J, Van Vugt J, Misajon R (2003). Developing a national index of subjective wellbeing: The Australian Unity Wellbeing Index. Social Indicators Research.

[CR13] Cummins RA, McCabe MP, Romeo Y, Gullone E (1994). The Comprehensive Quality-of-Life Scale (COMQOL)-instrument development and psychometric evaluation on college staff and students. Educational and Psychological Measurement.

[CR14] Cummins RA, Nistico H (2002). Maintaining life satisfaction: The role of positive cognitive bias. Journal of Happiness Studies.

[CR15] Cwik JC, Bussing A (2021). Spiritual needs of people with autism spectrum disorder. Spiritual needs in research and practice.

[CR16] Daley T (2002). The need for cross-cultural research on the pervasive developmental disorders. Transcultural Psychiatry.

[CR17] DaWalt LS, Taylor JL, Movaghar A, Hong J, Kim B, Brilliant M, Mailick MR (2021). Health profiles of adults with autism spectrum disorder: Differences between women and men. Autism Research.

[CR18] Dean M, Harwood R, Kasari C (2017). The art of camouflage: Gender differences in the social behaviors of girls and boys with autism spectrum disorder. Autism.

[CR105] Diener E (2000). Subjective well-being: The science of happiness and a proposal for a national index. American Psychologist.

[CR19] Draaisma D (2009). Stereotypes of autism. Philosophical Transactions of the Royal Society B: Biological Sciences.

[CR20] Duvekot J, van der Ende J, Verhulst FC, Slappendel G, van Daalen E, Maras A, Greaves-Lord K (2017). Factors influencing the probability of a diagnosis of autism spectrum disorder in girls versus boys. Autism.

[CR21] Dyches T, Wilder L, Sudweeks R, Obiakor F, Algozzine B (2004). Multicultural issues in autism. Journal of Autism and Developmental Disorders.

[CR22] Egilson ST, Olafsdottir LB, Leosdottir T, Saemundsen E (2017). Quality of life of high-functioning children and youth with autism spectrum disorder and typically developing peers: Self-and proxy-reports. Autism.

[CR23] Ennis-Cole D, Durodoye B, Harris H (2013). The impact of culture on autism diagnosis and treatment: Considerations for counselors and other professionals. The Family Journal.

[CR24] Fahs B (2020). There will be blood: Women’s positive and negative experiences with menstruation. Women’s Reproductive Health.

[CR25] Forjaz MJ, Prieto-Flores ME, Ayala A, Rodriguez-Blazquez C, Fernandez Mayoralas G, Rojo-Perez F, Martinez-Martin P (2011). Measurement properties of the community wellbeing Index in older adults. Quality of Life Research.

[CR26] Gardner M, Suplee PD, Bloch J, Lecks K (2016). Exploratory study of childbearing experiences of women with Asperger syndrome. Nursing for Women’s Health.

[CR27] Green RM, Travers AM, Howe Y, McDougle CJ (2019). Women and autism spectrum disorder: Diagnosis and implications for treatment of adolescents and adults. Current Psychiatry Reports.

[CR28] Griffiths S, Allison C, Kenny R, Holt R, Smith P, Baron-Cohen S (2019). The vulnerability experiences quotient (VEQ): A study of vulnerability, mental health and life satisfaction in autistic adults. Autism Research.

[CR29] Grinker RR, Yeargin-Allsopp M, Boyle C, Amaral D, Geschwind D, Dawson G (2011). Culture and autism spectrum disorders: The impact on prevalence and recognition. Autism spectrum disorders.

[CR30] Haney JL, Cullen JA (2017). Learning about the lived experiences of women with autism from an online community. Journal of Social Work in Disability & Rehabilitation.

[CR31] Helliwell J, Layard R, Sachs J (2018). World happiness report 2018.

[CR32] Hobson RP (2019). Autism and the development of mind.

[CR104] International Wellbeing Group (2013). Personal wellbeing index.

[CR33] Jamison TR, Schuttler JO (2015). Examining social competence, self-perception, quality of life, and internalizing and externalizing symptoms in adolescent females with and without autism spectrum disorder: A quantitative design including between-groups and correlational analyses. Molecular Autism.

[CR34] Kanfiszer L, Davies F, Collins S (2017). “I was just so different”: The experiences of women diagnosed with an autism spectrum disorder in adulthood in relation to gender and social relationships. Autism.

[CR35] Kang-Yi CD, Grinker RR, Mandell DS (2013). Korean culture and autism spectrum disorders. Journal of Autism and Developmental Disorders.

[CR36] Kenyon S (2014). Autism in pink European project: Qualitative research report.

[CR37] Kim HU (2012). Autism across cultures: Rethinking autism. Disability & Society.

[CR38] King LA, Hicks JA, Krull JL, Del Gaiso AK (2006). Positive affect and the experience of meaning in life. Journal of Personality and Social Psychology.

[CR101] La Placa, V., McNaught, A., & Knight, A. (2013). Discourse on wellbeing in research and practice. *International Journal of Wellbeing, 3*(1). 10.5502/ijw.v3i1.7

[CR39] Leedham A, Thompson AR, Smith R, Freeth M (2020). ‘I was exhausted trying to figure it out’: The experiences of females receiving an autism diagnosis in middle to late adulthood. Autism.

[CR40] Liu EX, Carter EW, Boehm TL, Annandale NH, Taylor CE (2014). In their own words: The place of faith in the lives of young people with autism and intellectual disability. Intellectual and Developmental Disabilities.

[CR41] Lonsdale S (1990). Women and disability.

[CR42] Lyubomirsky S, King L, Diener E (2005). The benefits of frequent positive affect: Does happiness lead to success?. Psychological Bulletin.

[CR44] Mason D, McConachie H, Garland D, Petrou A, Rodgers J, Parr JR (2018). Predictors of quality of life for autistic adults. Autism Research.

[CR45] Matson JL, Worley JA, Fodstad JC, Chung KM, Suh D, Jhin HK, Furniss F (2011). A multinational study examining the cross cultural differences in reported symptoms of autism spectrum disorders: Israel, South Korea, the UK and the USA. Research in Autism Spectrum Disorders.

[CR46] McDonald TAM (2020). Autism Identity and the “lost generation”: Structural validation of the autism spectrum identity scale and comparison of diagnosed and self-diagnosed adults on the autism spectrum. Autism in Adulthood.

[CR47] McGillivray JA, Evert HT (2018). Exploring the effect of gender and age on stress and emotional distress in adults with autism spectrum disorder. Focus on Autism and Other Developmental Disabilities.

[CR48] Milner V, Mcintosh H, Colvert E, Happe F (2019). A qualitative exploration of the female experience of autism spectrum disorder (ASD). Journal of Autism and Developmental Disorders.

[CR49] Moseley RL, Druce T, Turner-Cobb JM (2020). ‘When my autism broke’: A qualitative study spotlighting autistic voices on menopause. Autism.

[CR50] Moseley RL, Gregory NJ, Smith P, Allison C, Baron-Cohen S (2019). A “choice”, an “addiction”, a way “out of the lost”: Exploring self-injury in autistic people without intellectual disability. Molecular Autism.

[CR51] Mundy P, Hogan A (1994). Intersubjectivity, joint attention and autistic developmental pathology. Rochester symposium of developmental psychopathology.

[CR52] Obeid R (2015). A cross-cultural comparison of knowledge and stigma associated with autism spectrum disorder among college students in Lebanon and the USA. Jounral of Autism and Developmental Disorders.

[CR54] Oswald TM, Winter-Messiers MA, Gibson B, Schmidt AM, Herr CM, Solomon M (2015). Sex differences in internalizing problems during adolescence in autism spectrum disorder. Journal of Autism and Developmental Disorders.

[CR55] Pecora LA, Mesivov GB, Stokes MA (2016). Sexuality in high-functioning autism: a systematic review and meta-analysis. Journal of Autism and Developmental Disorders.

[CR56] Perepa P (2019). Autism, ethnicity and culture.

[CR57] Ravindran N, Myers BJ (2012). Cultural influences on perceptions of health, illness and disability: A review focus on autism. Journal of Child and Family Studies.

[CR58] Roberts AL, Koenen KC, Lyall K, Robinson EB, Weisskopf MG (2015). Association of autistic traits in adulthood with childhood abuse, interpersonal victimization, and posttraumatic stress. Child Abuse & Neglect.

[CR59] Rodriguez-Blazquez C, Frades-Payo B, Forjaz MJ, Ayala A, Martinez-Martin P, Fernandez-Mayoralas G, Rojo-Perez F (2011). Psychometric properties of the International Wellbeing Index in community-dwelling older adults. International Psychogeriatrics.

[CR103] Rogers C, Lepherd L, Ganguly R, Jacob-Rogers S (2017). Perinatal issues for women with high functioning autism spectrum disorder. Women and Birth.

[CR60] Schalock RL (2000). Three decades of quality of life. Focus on Autism and Other Developmental Disabilities.

[CR61] Shea NM, Millea MA, Diehl JJ (2013). Perceived autonomy support in children with autism spectrum disorder. Autism-Open Access.

[CR62] Solomon M, Miller M, Taylor SL, Hinshaw SP, Carter CS (2012). Autism symptoms and internalizing psychopathology in girls and boys with autism spectrum disorders. Journal of Autism and Developmental Disorders.

[CR63] Steward R, Crane L, Roy EM, Remington A, Pellicano E (2018). “Life is much more difficult to manage during periods”: Autistic experiences of menstruation. Journal of Autism and Developmental Disorders.

[CR64] Stodden RA, Mruzek DW (2010). An introduction to postsecondary education and employment of persons with autism and developmental disabilities. Focus on Autism and Other Developmental Disabilities.

[CR65] Sundelin HE, Stephansson O, Hultman CM, Ludvigsson JF (2018). Pregnancy outcomes in women with autism: A nationwide population-based cohort study. Clinical Epidemiology.

[CR66] Taylor JL, Smith DaWalt L, Marvin AR, Law JK, Lipkin P (2019). Sex differences in employment and supports for adults with autism spectrum disorder. Autism.

[CR67] Turner L (2017). Supporting women with autism during pregnancy, birth and beyond. MIDIRS Midwifery Digest.

[CR68] Van Heijst BF, Geurts HM (2015). Quality of life in autism across the lifespan: A meta-analysis. Autism.

[CR69] Van Wijngaarden-Cremers P, Barahona Corrêa B, van der Gaag RJ (2017). Autism in girls and women. Autism spectrum disorders in adults.

[CR70] Van Wijngaarden-Cremers P (2019). Autism in boys and girls, women and men throughout the lifespan. The Palgrave handbook of male psychology and mental health.

[CR71] Velikonja T, Fett AK, Velthorst E (2019). Patterns of nonsocial and social cognitive functioning in adults with autism spectrum disorder: A systematic review and meta-analysis. JAMA Psychiatry.

[CR72] Vermeulen P, Jones G, Hurley E (2014). The practice of promoting happiness in autism. Good autism practice: Autism, happiness and wellbeing.

[CR73] Vernon A (1997). The dialectics of multiple identities and the disabled people’s movement. Disability & Society.

[CR74] Wassell E, Dodge R (2015). A multidisciplinary framework for measuring and improving wellbeing. International Journal of Sciences: Basic and Applied Research (IJSBAR).

[CR75] Williams A (2018). Autonomously autistic. Canadian Journal of Disability Studies.

